# Closing the Loop: An Environmental Scan of APS-Reporter Feedback Policies and Practices

**DOI:** 10.1093/geroni/igab046.3370

**Published:** 2021-12-17

**Authors:** Olanike Ojelabi, Randi Campetti, Kathy Greenlee, Kristin Lees Haggerty

**Affiliations:** 1 Education Development Center, Waltham, Massachusetts, United States; 2 Greenlee Global LLC, Greenlee Global LLC, Kansas, United States; 3 Education Development Center, West Roxbury, Massachusetts, United States

## Abstract

Abuse, neglect, and exploitation of older adults are prevalent and underreported in the United States. Pathways to identifying and resolving cases of abuse against older adults depend on mandated and non-mandated reporters bringing attention to these cases through reports to Adult Protective Services (APS). However, existing research points to several barriers to reporting. One significant barrier is a lack of communication from APS to reporters about reports they have made (e.g., whether the report is appropriate for APS, the investigation outcome, and services provided by APS). This lack of reciprocal communication likely serves as a disincentive for future reporting. This study aims to promote improved communication between APS and reporters by examining the legal, ethical, and practical barriers and facilitators to communication at key points in the reporting and response pathways. In this first phase of the project, we conducted an environmental scan of policies and practices related to reporting, investigation, and feedback. Early results from the environmental scan suggest most APS agencies (81%) do not currently provide feedback to reporters. Among those providing feedback, 20% provide feedback only to mandated reporters, and 50% provide only procedural feedback, which focuses on the process of receiving and screening reports for investigation and not on the outcome of the investigation. In the next phase of this study, we will supplement these findings through interviews with APS leaders across the U.S. These early results will begin to fill an important gap in the understanding of feedback loops between APS and reporters.

